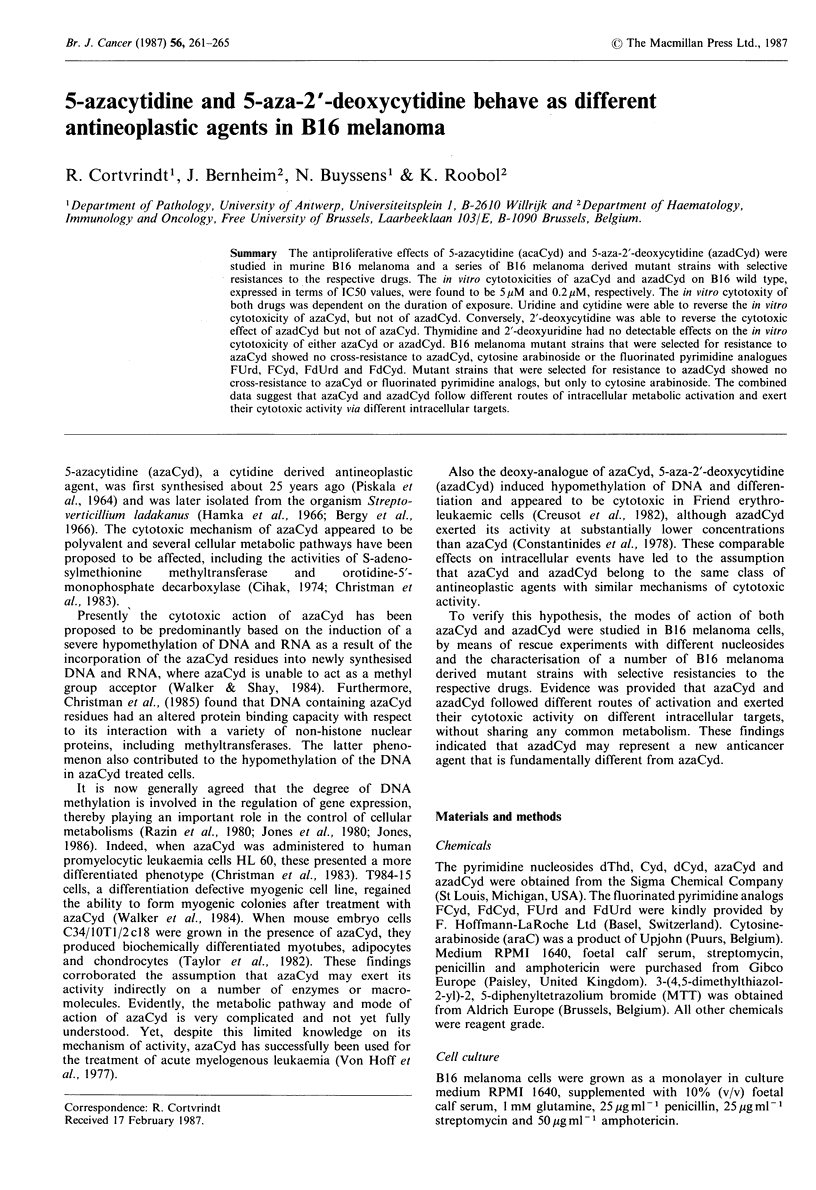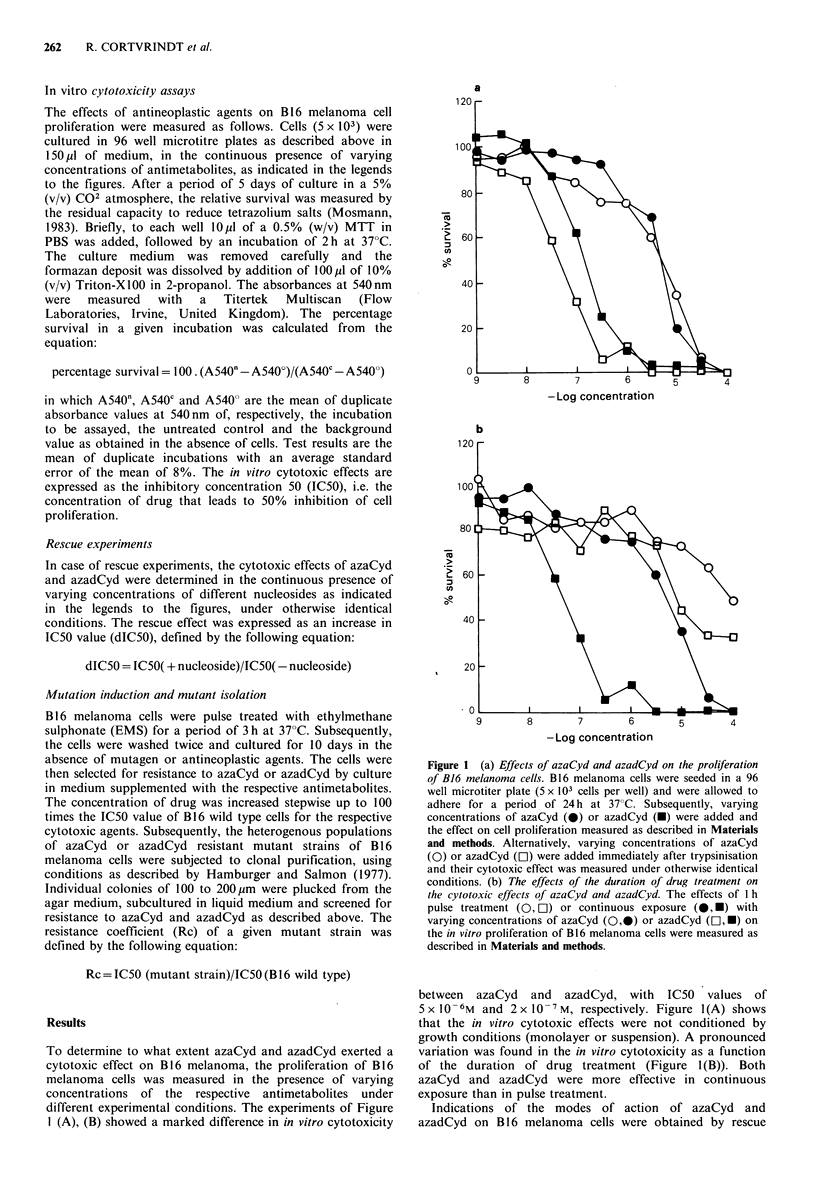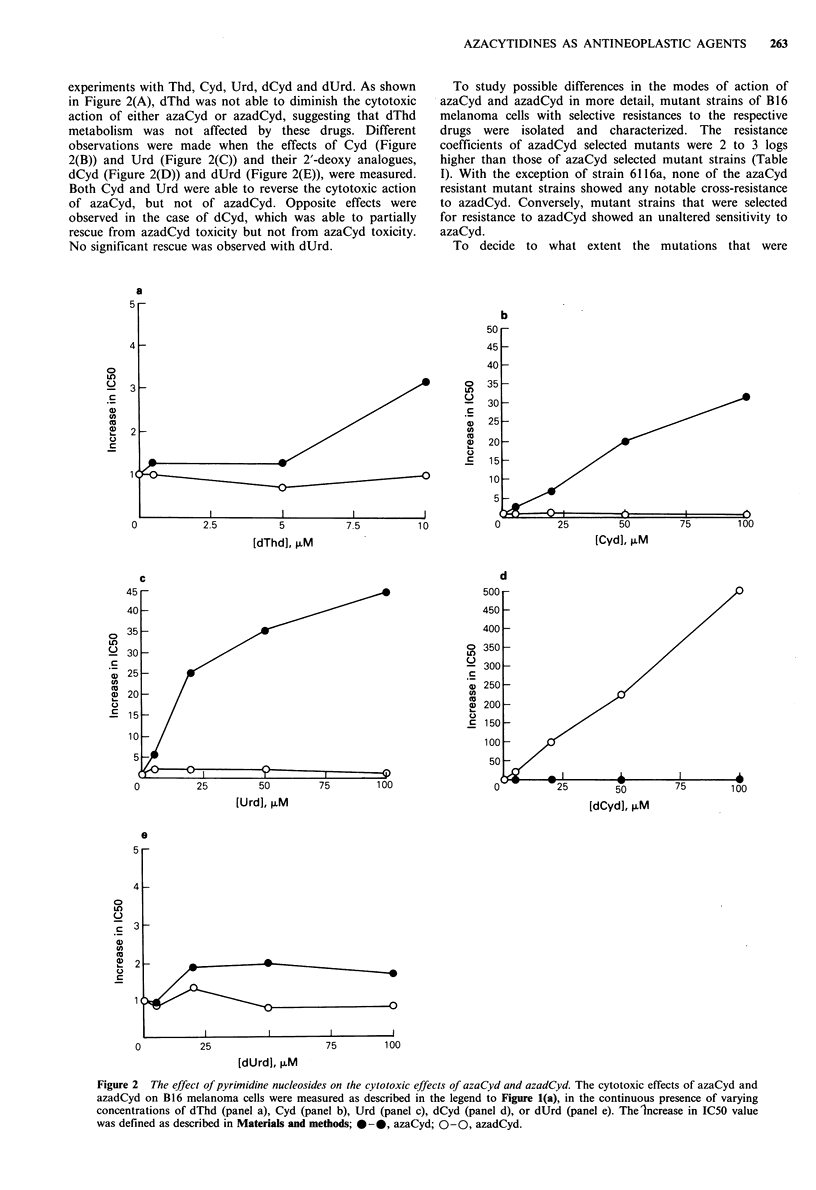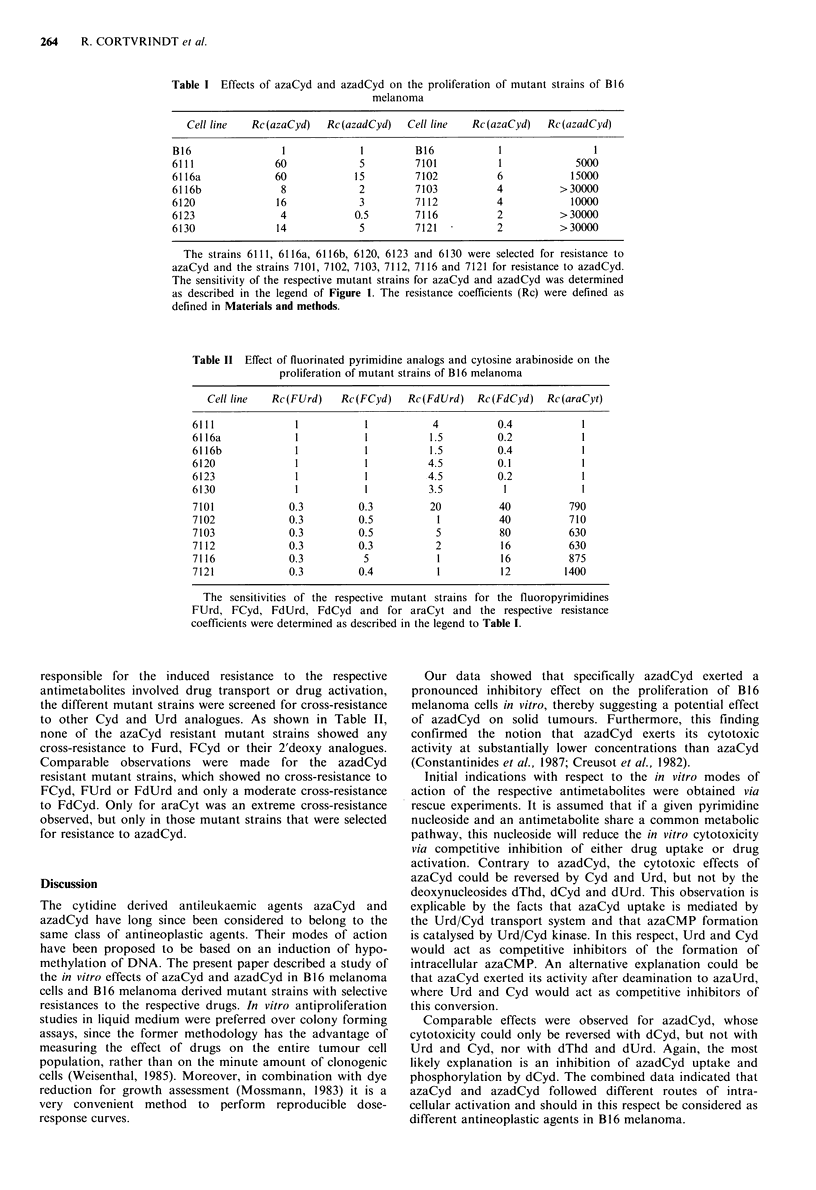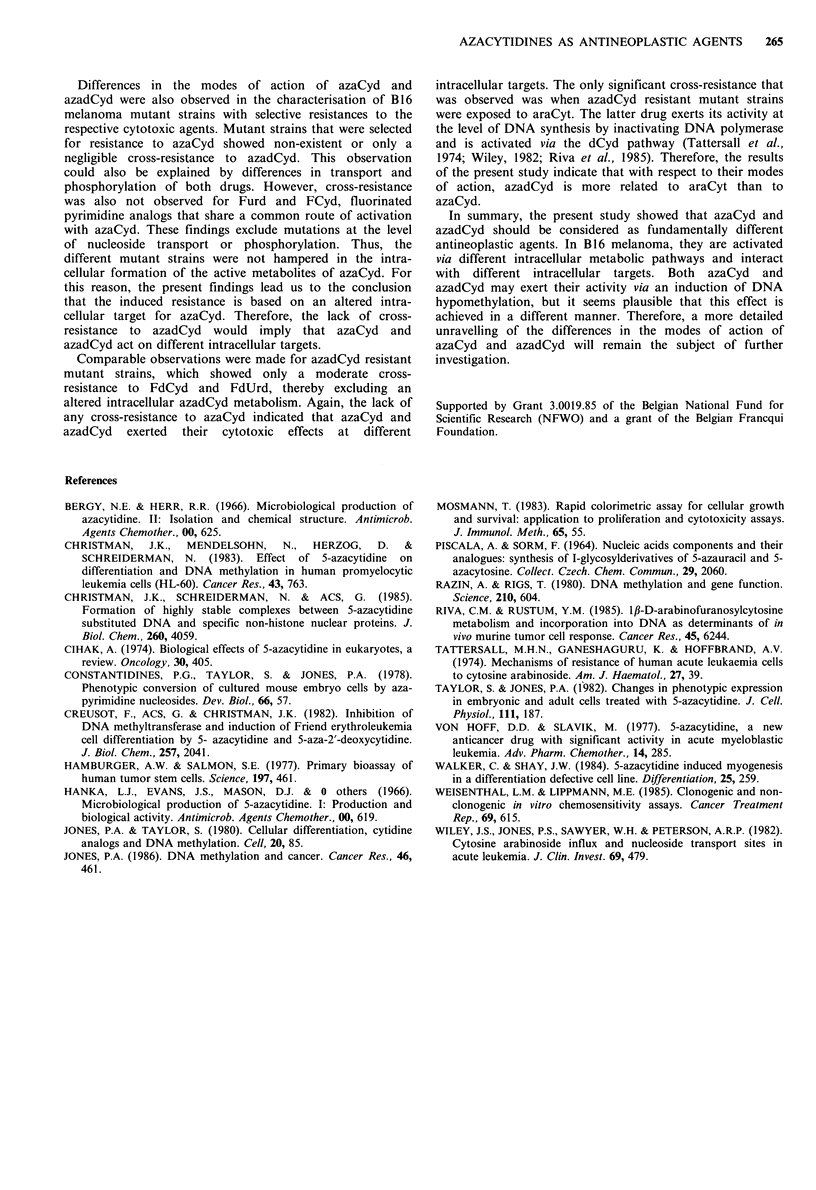# 5-Azacytidine and 5-aza-2'-deoxycytidine behave as different antineoplastic agents in B16 melanoma.

**DOI:** 10.1038/bjc.1987.187

**Published:** 1987-09

**Authors:** R. Cortvrindt, J. Bernheim, N. Buyssens, K. Roobol

**Affiliations:** Department of Pathology, University of Antwerp, Willrijk, Belgium.

## Abstract

The antiproliferative effects of 5-azacytidine (acaCyd) and 5-aza-2'-deoxycytidine (azadCyd) were studied in murine B16 melanoma and a series of B16 melanoma derived mutant strains with selective resistances to the respective drugs. The in vitro cytotoxicities of azaCyd and azadCyd on B16 wild type, expressed in terms of IC50 values, were found to be 5 microM and 0.2 microM, respectively. The in vitro cytotoxicity of both drugs was dependent on the duration of exposure. Uridine and cytidine were able to reverse the in vitro cytotoxicity of azaCyd, but not of azadCyd. Conversely, 2'-deoxycytidine was able to reverse the cytotoxic effect of azadCyd but not of azaCyd. Thymidine and 2'-deoxyuridine had no detectable effects on the in vitro cytotoxicity of either azaCyd or azadCyd. B16 melanoma mutant strains that were selected for resistance to azaCyd showed no cross-resistance to azadCyd, cytosine arabinoside or the fluorinated pyrimidine analogues FUrd, FCyd, FdUrd and FdCyd. Mutant strains that were selected for resistance to azadCyd showed no cross-resistance to azaCyd or fluorinated pyrimidine analogs, but only to cytosine arabinoside. The combined data suggest that azaCyd and azadCyd follow different routes of intracellular metabolic activation and exert their cytotoxic activity via different intracellular targets.


Br. J. Cancer (1987) 56, 261–265 © The Macmillan Press Ltd., 1987

### 5-azacytidine and 5-aza-2′-deoxycytidine behave as different antineoplastic agents in B16 melanoma

R. Cortvrindt[1], J. Bernheim[2], N. Buyssens[1] & K. Roobol[2]

[1]*Department of Pathology, University of Antwerp, Universiteitsplein 1, B-2610 Willrijk and* [2]*Department of Haematology, Immunology and Oncology, Free University of Brussels, Laarbeeklaan 103/E, B-1090 Brussels, Belgium.*

**Summary** The antiproliferative effects of 5-azacytidine (acaCyd) and 5-aza-2′-deoxycytidine (azadCyd) were studied in murine B16 melanoma and a series of B16 melanoma derived mutant strains with selective resistances to the respective drugs. The *in vitro* cytotoxicities of azaCyd and azadCyd on B16 wild type, expressed in terms of IC50 values, were found to be 5 $\mu$M and 0.2 $\mu$M, respectively. The *in vitro* cytotoxity of both drugs was dependent on the duration of exposure. Uridine and cytidine were able to reverse the *in vitro* cytotoxicity of azaCyd, but not of azadCyd. Conversely, 2′-deoxycytidine was able to reverse the cytotoxic effect of azadCyd but not of azaCyd. Thymidine and 2′-deoxyuridine had no detectable effects on the *in vitro* cytotoxicity of either azaCyd or azadCyd. B16 melanoma mutant strains that were selected for resistance to azaCyd showed no cross-resistance to azadCyd, cytosine arabinoside or the fluorinated pyrimidine analogues FUrd, FCyd, FdUrd and FdCyd. Mutant strains that were selected for resistance to azadCyd showed no cross-resistance to azaCyd or fluorinated pyrimidine analogs, but only to cytosine arabinoside. The combined data suggest that azaCyd and azadCyd follow different routes of intracellular metabolic activation and exert their cytotoxic activity *via* different intracellular targets.

5-azacytidine (azaCyd), a cytidine derived antineoplastic agent, was first synthesised about 25 years ago (Piskala *et al.*, 1964) and was later isolated from the organism *Streptoverticillium ladakanus* (Hamka *et al.*, 1966; Bergy *et al.*, 1966). The cytotoxic mechanism of azaCyd appeared to be polyvalent and several cellular metabolic pathways have been proposed to be affected, including the activities of S-adenosylmethionine methyltransferase and orotidine-5′-monophosphate decarboxylase (Cihak, 1974; Christman *et al.*, 1983).

Presently the cytotoxic action of azaCyd has been proposed to be predominantly based on the induction of a severe hypomethylation of DNA and RNA as a result of the incorporation of the azaCyd residues into newly synthesised DNA and RNA, where azaCyd is unable to act as a methyl group acceptor (Walker & Shay, 1984). Furthermore, Christman *et al.*, (1985) found that DNA containing azaCyd residues had an altered protein binding capacity with respect to its interaction with a variety of non-histone nuclear proteins, including methyltransferases. The latter phenomenon also contributed to the hypomethylation of the DNA in azaCyd treated cells.

It is now generally agreed that the degree of DNA methylation is involved in the regulation of gene expression, thereby playing an important role in the control of cellular metabolisms (Razin *et al.*, 1980; Jones *et al.*, 1980; Jones, 1986). Indeed, when azaCyd was administered to human promyelocytic leukaemia cells HL 60, these presented a more differentiated phenotype (Christman *et al.*, 1983). T984-15 cells, a differentiation defective myogenic cell line, regained the ability to form myogenic colonies after treatment with azaCyd (Walker *et al.*, 1984). When mouse embryo cells C34/10T1/2 cl8 were grown in the presence of azaCyd, they produced biochemically differentiated myotubes, adipocytes and chondrocytes (Taylor *et al.*, 1982). These findings corroborated the assumption that azaCyd may exert its activity indirectly on a number of enzymes or macromolecules. Evidently, the metabolic pathway and mode of action of azaCyd is very complicated and not yet fully understood. Yet, despite this limited knowledge on its mechanism of activity, azaCyd has successfully been used for the treatment of acute myelogenous leukaemia (Von Hoff *et al.*, 1977).

Correspondence: R. Cortvrindt
Received 17 February 1987.

Also the deoxy-analogue of azaCyd, 5-aza-2′-deoxycytidine (azadCyd) induced hypomethylation of DNA and differentiation and appeared to be cytotoxic in Friend erythroleukaemic cells (Creusot *et al.*, 1982), although azadCyd exerted its activity at substantially lower concentrations than azaCyd (Constantinides *et al.*, 1978). These comparable effects on intracellular events have led to the assumption that azaCyd and azadCyd belong to the same class of antineoplastic agents with similar mechanisms of cytotoxic activity.

To verify this hypothesis, the modes of action of both azaCyd and azadCyd were studied in B16 melanoma cells, by means of rescue experiments with different nucleosides and the characterisation of a number of B16 melanoma derived mutant strains with selective resistancies to the respective drugs. Evidence was provided that azaCyd and azadCyd followed different routes of activation and exerted their cytotoxic activity on different intracellular targets, without sharing any common metabolism. These findings indicated that azadCyd may represent a new anticancer agent that is fundamentally different from azaCyd.

### Materials and methods

### *Chemicals*

The pyrimidine nucleosides dThd, Cyd, dCyd, azaCyd and azadCyd were obtained from the Sigma Chemical Company (St Louis, Michigan, USA). The fluorinated pyrimidine analogs FCyd, FdCyd, FUrd and FdUrd were kindly provided by F. Hoffmann-LaRoche Ltd (Basel, Switzerland). Cytosine-arabinoside (araC) was a product of Upjohn (Puurs, Belgium). Medium RPMI 1640, foetal calf serum, streptomycin, penicillin and amphotericin were purchased from Gibco Europe (Paisley, United Kingdom). 3-(4,5-dimethylthiazol-2-yl)-2, 5-diphenyltetrazolium bromide (MTT) was obtained from Aldrich Europe (Brussels, Belgium). All other chemicals were reagent grade.

### *Cell culture*

B16 melanoma cells were grown as a monolayer in culture medium RPMI 1640, supplemented with 10% (v/v) foetal calf serum, 1 mM glutamine, 25 $\mu g\,ml^{-1}$ penicillin, 25 $\mu g\,ml^{-1}$ streptomycin and 50 $\mu g\,ml^{-1}$ amphotericin.

### *In vitro cytotoxicity assays*

The effects of antineoplastic agents on B16 melanoma cell proliferation were measured as follows. Cells ($5 \times 10^3$) were cultured in 96 well microtitre plates as described above in $150\,\mu l$ of medium, in the continuous presence of varying concentrations of antimetabolites, as indicated in the legends to the figures. After a period of 5 days of culture in a 5% (v/v) $CO^2$ atmosphere, the relative survival was measured by the residual capacity to reduce tetrazolium salts (Mosmann, 1983). Briefly, to each well $10\,\mu l$ of a 0.5% (w/v) MTT in PBS was added, followed by an incubation of 2 h at 37°C. The culture medium was removed carefully and the formazan deposit was dissolved by addition of $100\,\mu l$ of 10% (v/v) Triton-X100 in 2-propanol. The absorbances at 540 nm were measured with a Titertek Multiscan (Flow Laboratories, Irvine, United Kingdom). The percentage survival in a given incubation was calculated from the equation:

$$\text{percentage survival} = 100\,.\,(A540^{n} - A540^{o})/(A540^{c} - A540^{o})$$

in which $A540^n$, $A540^c$ and $A540^o$ are the mean of duplicate absorbance values at 540 nm of, respectively, the incubation to be assayed, the untreated control and the background value as obtained in the absence of cells. Test results are the mean of duplicate incubations with an average standard error of the mean of 8%. The *in vitro* cytotoxic effects are expressed as the inhibitory concentration 50 (IC50), i.e. the concentration of drug that leads to 50% inhibition of cell proliferation.

### *Rescue experiments*

In case of rescue experiments, the cytotoxic effects of azaCyd and azadCyd were determined in the continuous presence of varying concentrations of different nucleosides as indicated in the legends to the figures, under otherwise identical conditions. The rescue effect was expressed as an increase in IC50 value (dIC50), defined by the following equation:

$$\text{dIC50} = \text{IC50}(+\text{nucleoside})/\text{IC50}(-\text{nucleoside})$$

### *Mutation induction and mutant isolation*

B16 melanoma cells were pulse treated with ethylmethane sulphonate (EMS) for a period of 3 h at 37°C. Subsequently, the cells were washed twice and cultured for 10 days in the absence of mutagen or antineoplastic agents. The cells were then selected for resistance to azaCyd or azadCyd by culture in medium supplemented with the respective antimetabolites. The concentration of drug was increased stepwise up to 100 times the IC50 value of B16 wild type cells for the respective cytotoxic agents. Subsequently, the heterogenous populations of azaCyd or azadCyd resistant mutant strains of B16 melanoma cells were subjected to clonal purification, using conditions as described by Hamburger and Salmon (1977). Individual colonies of 100 to $200\,\mu m$ were plucked from the agar medium, subcultured in liquid medium and screened for resistance to azaCyd and azadCyd as described above. The resistance coefficient (Rc) of a given mutant strain was defined by the following equation:

$$\text{Rc} = \text{IC50 (mutant strain)}/\text{IC50 (B16 wild type)}$$

**Figure 1** (a) *Effects of azaCyd and azadCyd on the proliferation of B16 melanoma cells.* B16 melanoma cells were seeded in a 96 well microtiter plate ($5 \times 10^3$ cells per well) and were allowed to adhere for a period of 24 h at 37°C. Subsequently, varying concentrations of azaCyd (●) or azadCyd (■) were added and the effect on cell proliferation measured as described in **Materials and methods**. Alternatively, varying concentrations of azaCyd (○) or azadCyd (□) were added immediately after trypsinisation and their cytotoxic effect was measured under otherwise identical conditions. (b) *The effects of the duration of drug treatment on the cytotoxic effects of azaCyd and azadCyd.* The effects of 1 h pulse treatment (○, □) or continuous exposure (●, ■) with varying concentrations of azaCyd (○, ●) or azadCyd (□, ■) on the *in vitro* proliferation of B16 melanoma cells were measured as described in **Materials and methods**.

### Results

To determine to what extent azaCyd and azadCyd exerted a cytotoxic effect on B16 melanoma, the proliferation of B16 melanoma cells was measured in the presence of varying concentrations of the respective antimetabolites under different experimental conditions. The experiments of Figure 1 (A), (B) showed a marked difference in *in vitro* cytotoxicity between azaCyd and azadCyd, with IC50 values of $5 \times 10^{-6}$ M and $2 \times 10^{-7}$ M, respectively. Figure 1(A) shows that the *in vitro* cytotoxic effects were not conditioned by growth conditions (monolayer or suspension). A pronounced variation was found in the *in vitro* cytotoxicity as a function of the duration of drug treatment (Figure 1(B)). Both azaCyd and azadCyd were more effective in continuous exposure than in pulse treatment.

Indications of the modes of action of azaCyd and azadCyd on B16 melanoma cells were obtained by rescue experiments with Thd, Cyd, Urd, dCyd and dUrd. As shown in Figure 2(A), dThd was not able to diminish the cytotoxic action of either azaCyd or azadCyd, suggesting that dThd metabolism was not affected by these drugs. Different observations were made when the effects of Cyd (Figure 2(B)) and Urd (Figure 2(C)) and their 2′-deoxy analogues, dCyd (Figure 2(D)) and dUrd (Figure 2(E)), were measured. Both Cyd and Urd were able to reverse the cytotoxic action of azaCyd, but not of azadCyd. Opposite effects were observed in the case of dCyd, which was able to partially rescue from azadCyd toxicity but not from azaCyd toxicity. No significant rescue was observed with dUrd.

To study possible differences in the modes of action of azaCyd and azadCyd in more detail, mutant strains of B16 melanoma cells with selective resistances to the respective drugs were isolated and characterized. The resistance coefficients of azadCyd selected mutants were 2 to 3 logs higher than those of azaCyd selected mutant strains (Table I). With the exception of strain 6116a, none of the azaCyd resistant mutant strains showed any notable cross-resistance to azadCyd. Conversely, mutant strains that were selected for resistance to azadCyd showed an unaltered sensitivity to azaCyd.

To decide to what extent the mutations that were

**Figure 2** *The effect of pyrimidine nucleosides on the cytotoxic effects of azaCyd and azadCyd.* The cytotoxic effects of azaCyd and azadCyd on B16 melanoma cells were measured as described in the legend to **Figure 1(a)**, in the continuous presence of varying concentrations of dThd (panel a), Cyd (panel b), Urd (panel c), dCyd (panel d), or dUrd (panel e). The increase in IC50 value was defined as described in **Materials and methods**; ●–●, azaCyd; ○–○, azadCyd.

**Table I** Effects of azaCyd and azadCyd on the proliferation of mutant strains of B16 melanoma

| *Cell line* | *Rc(azaCyd)* | *Rc(azadCyd)* | *Cell line* | *Rc(azaCyd)* | *Rc(azadCyd)* |
|---|---|---|---|---|---|
| B16 | 1 | 1 | B16 | 1 | 1 |
| 6111 | 60 | 5 | 7101 | 1 | 5000 |
| 6116a | 60 | 15 | 7102 | 6 | 15000 |
| 6116b | 8 | 2 | 7103 | 4 | >30000 |
| 6120 | 16 | 3 | 7112 | 4 | 10000 |
| 6123 | 4 | 0.5 | 7116 | 2 | >30000 |
| 6130 | 14 | 5 | 7121 | 2 | >30000 |

The strains 6111, 6116a, 6116b, 6120, 6123 and 6130 were selected for resistance to azaCyd and the strains 7101, 7102, 7103, 7112, 7116 and 7121 for resistance to azadCyd. The sensitivity of the respective mutant strains for azaCyd and azadCyd was determined as described in the legend of **Figure 1**. The resistance coefficients (Rc) were defined as defined in **Materials and methods**.

**Table II** Effect of fluorinated pyrimidine analogs and cytosine arabinoside on the proliferation of mutant strains of B16 melanoma

| *Cell line* | *Rc(FUrd)* | *Rc(FCyd)* | *Rc(FdUrd)* | *Rc(FdCyd)* | *Rc(araCyt)* |
|---|---|---|---|---|---|
| 6111 | 1 | 1 | 4 | 0.4 | 1 |
| 6116a | 1 | 1 | 1.5 | 0.2 | 1 |
| 6116b | 1 | 1 | 1.5 | 0.4 | 1 |
| 6120 | 1 | 1 | 4.5 | 0.1 | 1 |
| 6123 | 1 | 1 | 4.5 | 0.2 | 1 |
| 6130 | 1 | 1 | 3.5 | 1 | 1 |
| 7101 | 0.3 | 0.3 | 20 | 40 | 790 |
| 7102 | 0.3 | 0.5 | 1 | 40 | 710 |
| 7103 | 0.3 | 0.5 | 5 | 80 | 630 |
| 7112 | 0.3 | 0.3 | 2 | 16 | 630 |
| 7116 | 0.3 | 5 | 1 | 16 | 875 |
| 7121 | 0.3 | 0.4 | 1 | 12 | 1400 |

The sensitivities of the respective mutant strains for the fluoropyrimidines FUrd, FCyd, FdUrd, FdCyd and for araCyt and the respective resistance coefficients were determined as described in the legend to **Table I**.

responsible for the induced resistance to the respective antimetabolites involved drug transport or drug activation, the different mutant strains were screened for cross-resistance to other Cyd and Urd analogues. As shown in Table II, none of the azaCyd resistant mutant strains showed any cross-resistance to Furd, FCyd or their 2′deoxy analogues. Comparable observations were made for the azadCyd resistant mutant strains, which showed no cross-resistance to FCyd, FUrd or FdUrd and only a moderate cross-resistance to FdCyd. Only for araCyt was an extreme cross-resistance observed, but only in those mutant strains that were selected for resistance to azadCyd.

### Discussion

The cytidine derived antileukaemic agents azaCyd and azadCyd have long since been considered to belong to the same class of antineoplastic agents. Their modes of action have been proposed to be based on an induction of hypomethylation of DNA. The present paper described a study of the *in vitro* effects of azaCyd and azadCyd in B16 melanoma cells and B16 melanoma derived mutant strains with selective resistances to the respective drugs. *In vitro* antiproliferation studies in liquid medium were preferred over colony forming assays, since the former methodology has the advantage of measuring the effect of drugs on the entire tumour cell population, rather than on the minute amount of clonogenic cells (Weisenthal, 1985). Moreover, in combination with dye reduction for growth assessment (Mossmann, 1983) it is a very convenient method to perform reproducible dose-response curves.

Our data showed that specifically azadCyd exerted a pronounced inhibitory effect on the proliferation of B16 melanoma cells *in vitro*, thereby suggesting a potential effect of azadCyd on solid tumours. Furthermore, this finding confirmed the notion that azadCyd exerts its cytotoxic activity at substantially lower concentrations than azaCyd (Constantinides *et al.*, 1987; Creusot *et al.*, 1982).

Initial indications with respect to the *in vitro* modes of action of the respective antimetabolites were obtained *via* rescue experiments. It is assumed that if a given pyrimidine nucleoside and an antimetabolite share a common metabolic pathway, this nucleoside will reduce the *in vitro* cytotoxicity *via* competitive inhibition of either drug uptake or drug activation. Contrary to azadCyd, the cytotoxic effects of azaCyd could be reversed by Cyd and Urd, but not by the deoxynucleosides dThd, dCyd and dUrd. This observation is explicable by the facts that azaCyd uptake is mediated by the Urd/Cyd transport system and that azaCMP formation is catalysed by Urd/Cyd kinase. In this respect, Urd and Cyd would act as competitive inhibitors of the formation of intracellular azaCMP. An alternative explanation could be that azaCyd exerted its activity after deamination to azaUrd, where Urd and Cyd would act as competitive inhibitors of this conversion.

Comparable effects were observed for azadCyd, whose cytotoxicity could only be reversed with dCyd, but not with Urd and Cyd, nor with dThd and dUrd. Again, the most likely explanation is an inhibition of azadCyd uptake and phosphorylation by dCyd. The combined data indicated that azaCyd and azadCyd followed different routes of intracellular activation and should in this respect be considered as different antineoplastic agents in B16 melanoma.

Differences in the modes of action of azaCyd and azadCyd were also observed in the characterisation of B16 melanoma mutant strains with selective resistances to the respective cytotoxic agents. Mutant strains that were selected for resistance to azaCyd showed non-existent or only a negligible cross-resistance to azadCyd. This observation could also be explained by differences in transport and phosphorylation of both drugs. However, cross-resistance was also not observed for Furd and FCyd, fluorinated pyrimidine analogs that share a common route of activation with azaCyd. These findings exclude mutations at the level of nucleoside transport or phosphorylation. Thus, the different mutant strains were not hampered in the intracellular formation of the active metabolites of azaCyd. For this reason, the present findings lead us to the conclusion that the induced resistance is based on an altered intracellular target for azaCyd. Therefore, the lack of cross-resistance to azadCyd would imply that azaCyd and azadCyd act on different intracellular targets.

Comparable observations were made for azadCyd resistant mutant strains, which showed only a moderate cross-resistance to FdCyd and FdUrd, thereby excluding an altered intracellular azadCyd metabolism. Again, the lack of any cross-resistance to azaCyd indicated that azaCyd and azadCyd exerted their cytotoxic effects at different intracellular targets. The only significant cross-resistance that was observed was when azadCyd resistant mutant strains were exposed to araCyt. The latter drug exerts its activity at the level of DNA synthesis by inactivating DNA polymerase and is activated *via* the dCyd pathway (Tattersall *et al.*, 1974; Wiley, 1982; Riva *et al.*, 1985). Therefore, the results of the present study indicate that with respect to their modes of action, azadCyd is more related to araCyt than to azaCyd.

In summary, the present study showed that azaCyd and azadCyd should be considered as fundamentally different antineoplastic agents. In B16 melanoma, they are activated *via* different intracellular metabolic pathways and interact with different intracellular targets. Both azaCyd and azadCyd may exert their activity *via* an induction of DNA hypomethylation, but it seems plausible that this effect is achieved in a different manner. Therefore, a more detailed unravelling of the differences in the modes of action of azaCyd and azadCyd will remain the subject of further investigation.

Supported by Grant 3.0019.85 of the Belgian National Fund for Scientific Research (NFWO) and a grant of the Belgian Francqui Foundation.

### References

BERGY, N.E. & HERR, R.R. (1966). Microbiological production of azacytidine. II: Isolation and chemical structure. *Antimicrob. Agents Chemother.*, **00**, 625.

CHRISTMAN, J.K., MENDELSOHN, N., HERZOG, D. & SCHREIDERMAN, N. (1983). Effect of 5-azacytidine on differentiation and DNA methylation in human promyelocytic leukemia cells (HL-60). *Cancer Res.*, **43**, 763.

CHRISTMAN, J.K., SCHREIDERMAN, N. & ACS, G. (1985). Formation of highly stable complexes between 5-azacytidine substituted DNA and specific non-histone nuclear proteins. *J. Biol. Chem.*, **260**, 4059.

CIHAK, A. (1974). Biological effects of 5-azacytidine in eukaryotes, a review. *Oncology*, **30**, 405.

CONSTANTIDINES, P.G., TAYLOR, S. & JONES, P.A. (1978). Phenotypic conversion of cultured mouse embryo cells by aza-pyrimidine nucleosides. *Dev. Biol.*, **66**, 57.

CREUSOT, F., ACS, G. & CHRISTMAN, J.K. (1982). Inhibition of DNA methyltransferase and induction of Friend erythroleukemia cell differentiation by 5- azacytidine and 5-aza-2′-deoxycytidine. *J. Biol. Chem.*, **257**, 2041.

HAMBURGER, A.W. & SALMON, S.E. (1977). Primary bioassay of human tumor stem cells. *Science*, **197**, 461.

HANKA, L.J., EVANS, J.S., MASON, D.J. & 0 others (1966). Microbiological production of 5-azacytidine. I: Production and biological activity. *Antimicrob. Agents Chemother.*, **00**, 619.

JONES, P.A. & TAYLOR, S. (1980). Cellular differentiation, cytidine analogs and DNA methylation. *Cell*, **20**, 85.

JONES, P.A. (1986). DNA methylation and cancer. *Cancer Res.*, **46**, 461.

MOSMANN, T. (1983). Rapid colorimetric assay for cellular growth and survival: application to proliferation and cytotoxicity assays. *J. Immunol. Meth.*, **65**, 55.

PISCALA, A. & SORM, F. (1964). Nucleic acids components and their analogues: synthesis of I-glycosylderivatives of 5-azauracil and 5-azacytosine. *Collect. Czech. Chem. Commun.*, **29**, 2060.

RAZIN, A. & RIGS, T. (1980). DNA methylation and gene function. *Science*, **210**, 604.

RIVA, C.M. & RUSTUM, Y.M. (1985). 1β-D-arabinofuranosylcytosine metabolism and incorporation into DNA as determinants of *in vivo* murine tumor cell response. *Cancer Res.*, **45**, 6244.

TATTERSALL, M.H.N., GANESHAGURU, K. & HOFFBRAND, A.V. (1974). Mechanisms of resistance of human acute leukaemia cells to cytosine arabinoside. *Am. J. Haematol.*, **27**, 39.

TAYLOR, S. & JONES, P.A. (1982). Changes in phenotypic expression in embryonic and adult cells treated with 5-azacytidine. *J. Cell. Physiol.*, **111**, 187.

VON HOFF, D.D. & SLAVIK, M. (1977). 5-azacytidine, a new anticancer drug with significant activity in acute myeloblastic leukemia. *Adv. Pharm. Chemother.*, **14**, 285.

WALKER, C. & SHAY, J.W. (1984). 5-azacytidine induced myogenesis in a differentiation defective cell line. *Differentiation*, **25**, 259.

WEISENTHAL, L.M. & LIPPMANN, M.E. (1985). Clonogenic and non-clonogenic *in vitro* chemosensitivity assays. *Cancer Treatment Rep.*, **69**, 615.

WILEY, J.S., JONES, P.S., SAWYER, W.H. & PETERSON, A.R.P. (1982). Cytosine arabinoside influx and nucleoside transport sites in acute leukemia. *J. Clin. Invest.* **69**, 479.